# Genetic screening of 202 individuals with congenital limb malformations and requiring reconstructive surgery

**DOI:** 10.1136/jmg.2009.066027

**Published:** 2009-05-07

**Authors:** D Furniss, S-h Kan, I B Taylor, D Johnson, P S Critchley, H P Giele, A O M Wilkie

**Affiliations:** 1 Weatherall Institute of Molecular Medicine, University of Oxford, Oxford, UK; 2 Department of Plastic and Reconstructive Surgery, John Radcliffe Hospital, Oxford, UK

## Abstract

**Background:**

Congenital limb malformations (CLMs) are common and present to a variety of specialties, notably plastic and orthopaedic surgeons, and clinical geneticists. The authors aimed to characterise causative mutations in an unselected cohort of patients with CLMs requiring reconstructive surgery.

**Methods:**

202 patients presenting with CLM were recruited. The authors obtained G-banded karyotypes and screened *EN1*, *GLI3*, *HAND2*, *HOXD13*, *ROR2*, *SALL1*, *SALL4*, *ZRS* of *SHH*, *SPRY4*, *TBX5*, *TWIST1* and *WNT7A* for point mutations using denaturing high performance liquid chromatography (DHPLC) and direct sequencing. Multiplex ligation dependent probe amplification (MLPA) kits were developed and used to measure copy number in *GLI3*, *HOXD13*, *ROR2*, *SALL1*, *SALL4,* *TBX5* and the *ZRS* of *SHH*.

**Results:**

Within the cohort, causative genetic alterations were identified in 23 patients (11%): mutations in *GLI3* (n = 5), *HOXD13* (n = 5), the *ZRS* of *SHH* (n = 4), and chromosome abnormalities (n = 4) were the most common lesions found. Clinical features that predicted the discovery of a genetic cause included a bilateral malformation, positive family history, and having increasing numbers of limbs affected (all p<0.01). Additionally, specific patterns of malformation predicted mutations in specific genes.

**Conclusions:**

Based on higher mutation prevalence the authors propose that *GLI3, HOXD13* and the *ZRS* of *SHH* should be prioritised for introduction into molecular genetic testing programmes for CLM. The authors have developed simple criteria that can refine the selection of patients by surgeons for referral to clinical geneticists. The cohort also represents an excellent resource to test for mutations in novel candidate genes.

Congenital limb malformations (CLMs) affect approximately 1 in 500 live births^1^ and usually require surgical intervention to improve functional and aesthetic outcome. CLMs are very diverse in their epidemiology, aetiology and anatomy. In around half of cases, CLMs occur bilaterally, and in unilateral CLM the right and left sides are affected with approximately equal frequency. Up to 18% of children with a CLM die before the age of 6 years, usually because of associated malformations.^1^ Major causes of CLM include intrauterine disruptions (for example, caused by fetal haemorrhage, hypovolaemia or teratogenesis) and genetic abnormalities (chromosome abnormalities and single gene mutations). Major anatomical categories of CLM include limb hypoplasia reduction defects, brachydactylies, and the polydactyly–syndactyly–triphalangism group.^2^

Previous investigations into the genetics of human CLM have taken two approaches. First, positional candidate methods have been used to identify mutated genes in affected families following linkage analysis, or in individuals harbouring chromosome abnormalities.^3^ ^4^ Second, genetic mutations causing CLM have been identified in model organisms such as the mouse, and the orthologous gene in humans has been screened for mutations in patients with a similar phenotype.^5^ Although these approaches have yielded many important gene discoveries, they also have inherent methodological problems. A suitable pedigree structure is required for linkage analysis, meaning that only a small proportion of patients will be suitable for study; similarly, chromosome abnormalities are a relatively uncommon cause of CLM. The model organism/candidate gene approach assumes a correlation between the phenotypes present in the model organism and in the human: this is not always the case. For example, heterozygous mutations in the mouse gene *Alx4* cause polydactyly, whereas the limbs are normal when the exactly equivalent mutation occurs in the human orthologue.^6^ ^7^

In this study we took a different approach to the genetic analysis of CLM. We recruited from a paediatric hand surgery clinic a large cohort of unselected patients with CLM, who underwent surgery; we then undertook mutation analysis of selected genes in the entire cohort, regardless of phenotype. This approach has three potential advantages. First, it makes no prior assumption about which patients will harbour mutations in which gene, allowing the discovery of novel phenotypes associated with mutations in genes already known to cause human CLM. Second, it allows unbiased estimation of the relative contribution of mutations in different genes to the total burden of human CLM; this has not been reported previously. Third, the cohort constitutes a large panel to search for mutations in novel candidate genes as they are discovered in model organisms, without assuming any specific phenotypic correlation in the pattern of CLM.

For this study we chose candidate genes falling into two broad groups. First, we selected genes in which mutations causing human CLMs had been described previously: *GLI3*, *HOXD13*, *ROR2*, *SALL1*, *SALL4*, *ZRS* of *SHH*, and *TBX5*.^3^ ^4^ ^5^ ^8^ ^9^ ^10^ ^11^ ^12^ ^13^ Here we aimed to quantify their contribution to human CLM and, potentially, to extend the phenotypic spectrum of mutations. Second, we selected genes either known to play critical roles in limb development or mutated in association with mouse CLMs, but for which mutations in the human orthologue causing isolated CLM had not at the time been reported: *EN1*, *HAND2*, *SPRY4*, *TWIST1*, and *WNT7A*.^14^ ^15^ ^16^ ^17^ Here, the aim was to describe novel mutations and therefore provide further insight into the molecular genetics of human limb formation and CLM.

In this report, we describe this genetic analysis of the cohort. The results were also used to identify clinical characteristics that predict a genetic aetiology, and thereby define referral criteria for patients to clinical genetics services.

## Methods

### Subjects

Approval for the work was obtained from the Oxford Research Ethics Committee C (C99.181: Molecular basis of congenital limb abnormalities). Consent was requested from all parents/guardians of patients presenting between 1999 and 2006 to the Department of Plastic and Reconstructive Surgery, Oxford, with a congenital CLM requiring reconstructive surgery. At operation, 3–10 ml of venous blood was collected, which was used for routine karyotype analysis and isolation of genomic DNA using phenol/chloroform extraction. A database containing detailed phenotypic information on each patient recruited to the study was created, and the clinical notes of all patients were individually reviewed.

The family history was obtained by the surgeon in clinic. For statistical comparisons, we utilised two definitions of a positive family history of CLM: (1) having any relative affected with an identical or very similar CLM; and (2) the more strict definition of having an affected first degree relative.

### Mutation screening

Screening for point mutations was undertaken by WAVE denaturing high performance liquid chromatography (DHPLC) (Transgenomic, Omaha, Nebraska, USA), followed by direct sequencing of abnormally eluting fragments as previously described.^18^ All primer sequences and reaction conditions are available on request.^19^ Owing both to continuing recruitment while molecular analysis was ongoing, and mutations being discovered in some patients, each gene was screened in different numbers of patients as follows: *EN1*, 187; *GLI3*, 198; *HAND2*, 174; *HOXD13*, 175; *ROR2*, 139; *SALL1*, 197; *SALL4*, 183; *ZRS* of *SHH*, 187; *SPRY4*, 149; *TBX5*, 160; *TWIST1*, 188; *WNT7A*, 187.

In collaboration with MRC-Holland (Amsterdam, The Netherlands), we designed^20^ multiplex ligation dependent probe amplification (MLPA) probe sets to test for deletions of all exons of *GLI3, HOXD13, ROR2, TBX5, SALL1* and *SALL4* (MLPA probe sets P179 and P180, MRC-Holland). Details of the probe sequence at the ligation site are provided in supplemental tables S1 and S2. The probe sets P179 and P180 were used to screen 194 and 198 subjects, respectively. A subset of patients (n = 26), chosen because they had bilateral syndactyly and/or polydactyly, was screened for abnormal dosage of the *ZRS* of *SHH* using a previously described MLPA probe mix.^21^ Statistical comparisons between dichotomous variables were made using Fisher’s exact test.

## Results

### Genetic abnormalities identified in the cohort

In total, 202 patients were recruited to the study; their clinical features are summarised in table 1. Of these, 98 (49%) had more than one limb affected; 51 (25%) had a family history of CLM including 42 (21%) with an affected first degree relative. Twenty-seven patients (13%) had non-limb malformations, which in 13 (6%) constituted a recognised syndrome or association. The most common CLM was polydactyly (56% of cases), with postaxial being about twice as common as preaxial polydactyly. Syndactyly, either isolated or combined with polydactyly, was the next most common malformation (21%), followed by longitudinal dysplasia (9%) and symbrachydactyly (6%).

**Table 1:** Clinical characteristics of the cohort

Phenotypic characteristic	Number*	% Mutation positive	% Genetic component†
Total	202	11	32
Number of limbs affected			
4	20	30	55
3	6	33	50
2	72	17	44
1	104	2	17
Bilateral malformation	97	22	47
Positive family history	51	24	100
Syndrome or association	13	38	54
Other non-limb malformations not classified into a syndrome	14	36	43
Polydactyly			
Postaxial	75	5	39
Preaxial	34	26	38
Pre- and postaxial	4	50	50
Triphalangeal thumb	5	80	80
Syndactyly	31	16	32
Polysyndactyly	12	33	42
Longitudinal dysplasia			
Radial	8	25	63
Ulnar	3	0	0
Central	7	0	14
Symbrachydactyly	13	0	0
Brachydactyly	1	0	100
Clinodactyly	8	13	25
Camptodactyly	4	25	25
Hypoplastic digits			
Thumb	4	0	0
Other digits	4	0	0
Trigger thumb	5	0	0
Constriction ring syndrome	3	0	0
Other	10	30	30

*Totals do not add up to 202 as 24 children had multiple limb malformations which were included in between two and five categories.

†Genetic component consists of individuals with either a cytogenetic or molecularly proven diagnosis, a positive family history of congenital limb malformation (CLM), or a known genetically determined syndrome (see section: Estimation of the overall genetic contribution to CLM).

The cohort includes five patients in whom a cytogenetic or molecular diagnosis (three chromosome abnormalities, and single mutations in *ESCO2* and *SALL1*) was made independently as a result of routine clinical care. In a further 18 cases, a new cytogenetic or molecular diagnosis was obtained through our research protocol, giving a total of 23 subjects (11% of the total) with a proven genetic lesion accounting for their malformation. Several of the mutations in *GLI3*, *HOXD13, SALL1*, and *ZRS* of *SHH* discovered in this cohort have been reported in previous publications^18^ ^21^ ^22^ ^23^; selected data for unpublished mutations are shown in supplemental fig S1.

The molecular/cytogenetic and clinical details of this “genetic diagnosis” cohort are summarised in table 2. Of the 19 molecular alterations listed in table 2, 13 were considered obviously pathogenic because they represented deletions, duplications or nonsense mutations involving known disease genes. Of the remaining nucleotide substitutions, supporting evidence for pathogenicity of the 940A>C (I314L) substitution encoded by *HOXD13* and the 295T>C substitution in the *ZRS* of *SHH* is discussed elsewhere.^18^ ^21^ The 266T>A mutation in *TBX5*, encoding V89E, affects a conserved residue in the DNA binding T-box and was not identified in 265 controls.

**Table 2:** Clinical characteristics of patients with a confirmed molecular genetic diagnosis

Gene	Patient number	Mutation (heterozygous unless stated otherwise)	Clinical features*			Bilateral	Family history	Number of limbs affected	Final diagnosis	Reference
			Hands	Feet	Other					
*GLI3*	OX1746	366C>G, Y122X	Synd 3rd web L	PrP, Synd 1st 2nd 3rd webs	Macrocephaly	Yes	Yes	3	GCPS	–
*GLI3*	OX2879	1320dupT, E441X	PrP, PoP type B	PrP, Synd 1st 2nd webs	Macrocephaly, undescended testicle	Yes	No	4	GCPS	22
*GLI3*	OX2877†	2372delC, P791RfsX3	PoP type B	–	–	Yes	Yes	2	PoP type A1	22
*GLI3*	OX3536	2374C>T, R792X	PoP type B	PrP	Hypertelorism	Yes	Yes	4	GCPS	22
*GLI3*	OX3448	Deletion exons 10–14	PoP, Synd 2nd 3rd webs R hand, Synd 3rd 4th webs L hand	PrP, Synd 1st 2nd webs	–	Yes	Yes	4	GCPS	–
*HOXD13*	OX2137	165_185dup, A55_A61dup	Synd 3rd web	PoP, Synd 4th web	–	Yes	Yes	4	SPD1	18
*HOXD13*	OX1928	752-2delA	Synd 3rd web R hand, clinodactyly little fingers	Extra bony element in 1st web space	–	Yes	Yes	3	SPD1 with foot anomaly	23
*HOXD13*	OX1749	940A>C, I314L	Lateral duplication of ring finger phalanges, Synd 3rd webs	–	–	Yes	Yes	2	SPD1/brachydactyly E overlap	18
*HOXD13*	OX1752	940A>C, I314L	Little finger hypoplasia, lateral duplication of ring finger phalanges	–	–	Yes	Yes	2	SPD1/brachydactyly E overlap	18
*HOXD13*	OX3015†	955C>T, R319X	Clinodactyly little fingers	PoP	–	Yes	Yes	3	CLM with *HOXD13* mutation	–
*SALL1*	OX3335§	995delC, P332HfsX10	PrP, R side Wassel type 6, L side type 3	–	Imperforate anus, rectal atresia, hypospadias, overfolded helices	Yes	No	2	Townes-Brocks syndrome	22, 30
*SALL1*	OX2948†	3414_3415delAT, C1139WfsX14	PrP R, TpT R	–	–	No	No	1	CLM with *SALL1* mutation	22
*SALL4*	OX3701	2593C>T, R865X	Hypoplastic thumbs, L side Blauth type 3a, R side type 3b	–	Anal stenosis, ventriculo-septal defect, vascular malformation	Yes	Yes	2	Okihiro syndrome	–
*TBX5*	OX2084†	266T>A, V89E	Grade 1 radial dysplasia with hypoplastic thumbs, Blauth type 4 R hand and type 5 L hand	–	–	Yes	No	2	Holt-Oram syndrome	–
*ZRS*	OX1925	295T>C	TpT	–	–	Yes	No	2	TpT with *ZRS* mutation	21
*ZRS*	OX3159	295T>C	TpT, PrP	–	–	Yes	Yes	2	PrP type II	21
*ZRS*	OX3601	295T>C	TpT	–	–	Yes	Yes	2	TpT with *ZRS* mutation	21
*ZRS*	OX3424	Triplication	Complex polysyndactyly, fixed flexion at wrists	Complex polysyndactyly, severe talipes, mirror L foot	Closed spina bifida	Yes	No	4	Synd type IV (Haas)	–
–	OX2612§	t(2;18)(q14.2;p11.2)	Oligodactyly, more severe radially	Dislocated patellae, tibial shortening, fibular bowing, single digit	–	Yes	No	4	Split-hand/foot malformation with long bone deficiency 1	26
–	OX3689†‡	dup(6)(p22.2p23)	PrP	–	Low birthweight, microcephaly, developmental delay	Yes	No	2	CLM with chromosome abnormality	–
–	OX3084§	del(22)(q11.2q11.2)	PrP	–	Cardiac malformation	No	No	1	22q11.2 deletion syndrome	–
–	OX3126§	del(9)(p22.1)	Camptodactyly, thumb hypoplasia	–	–	Yes	No	2	9p deletion syndrome	–
*ESCO2*	OX3470§	Homozygous, 955+2_+5delTAAG	Radial dysplasia	Talipes equinovarus	Micrognathia, long columella, hypoplastic alae, nasal haemangioma, macrocephaly.	Yes	No	2	Roberts syndrome	31

CLM, congenital limb malformation; GCPS, Greig cephalopolysyndactyly syndrome; L, left sided; PoP, postaxial polydactyly; PrP, preaxial polydactyly; R, right sided; Synd, syndactyly; SPD1, synpolydactyly 1; TpT, triphalangeal thumb.

*Limbs were bilaterally affected unless otherwise stated.

†Genetic abnormality that would not have been discovered by a clinically focused approach to mutation screening (see Discussion).

‡Cytogenetic diagnosis made as part of the study protocol.

§Clinical diagnosis made after consultation with clinical genetics service. Molecular genetic or cytogenetic diagnosis was made outside of this study.

The most common causative genetic alterations that we identified in our CLM cohort were heterozygous mutations in *GLI3* (n = 5), *HOXD13* (n = 5), *ZRS* of *SHH* (n = 4), and miscellaneous microscopically visible chromosome abnormalities (n = 4). More unusual were mutations in *SALL1* (n = 2), *SALL4* (n = 1) and *TBX5* (n = 1). We did not find any pathogenic mutations in the remaining genes screened, including all those genes not yet associated with defined human CLM syndromes (*EN1*, *HAND2*, *SPRY4* and *TWIST1*), as well as *ROR2* and *WNT7A*. After this study was initiated, recessive mutations of *WNT7A* were reported in Al-Awadi/Raas-Rothschild/Schinzel phocomelia and Fuhrmann syndromes (MIM 228930)^24^; however, our cohort did not include any individuals with these disorders.

In addition to the above pathogenic mutations, we identified 33 additional non-synonymous variants that were considered either non-pathogenic or where the evidence was inconclusive, as itemised in supplemental table S3. A further 80 synonymous and non-coding variants were identified: where these were not known single nucleotide polymorphisms (SNPs), we checked the possibility that they created cryptic splice sites using a neural network splice site prediction program (supplemental tables S4–S6). Our sample size is too small to exclude the possibility that some of these variants act as susceptibility alleles for particular CLMs.

### Factors predicting the discovery of a genetic cause for the CLM

We examined both the general clinical features of the 23 subjects in the genetic diagnosis group and the specific clinical features that might have led to the correct genetic diagnosis independently of our research protocol. Twenty-one of the 23 patients had a bilateral malformation (96%), compared to 76/179 (42%) without a confirmed genetic diagnosis (p<5×10^−6^). There was a positive family history of CLM in 12/23 (52%) with a genetic diagnosis, compared to 39/179 (22%) without (p = 0.004). Using the stricter criterion of having a first degree relative affected with an identical or very similar malformation, 12/23 (52%) with a genetic diagnosis had a positive family history of CLM, compared to 30/179 (17%) without (p = 0.0004). Having increasing numbers of limbs affected also predicted the discovery of a molecular genetic cause for the malformation: 6/23 (26%) of those with a molecular genetic diagnosis had all four limbs affected, compared to 12/179 (7%) without (p = 0.008), and 21/23 (91%) of patients with a molecular genetic diagnosis had more than one limb affected, compared to 77/179 (43%) without (p = 6×10^−6^).

Specific patterns of malformation were also associated with the discovery of a mutation. Four out of five patients (80%) with mutations in *GLI3* had a combination of bilateral preaxial polydactyly of the feet and a hand malformation. In contrast, only two other patients had preaxial polydactyly of the foot, in both cases it was unilateral, and in only one case was it associated with a hand malformation. Thus, the presence of bilateral preaxial polydactyly of the feet, especially if combined with a hand malformation, is strongly associated with mutation in *GLI3*.

Two other examples of specific patterns of malformation are provided by triphalangeal thumb and ring finger duplication. All three patients with bilateral triphalangeal thumb harboured an identical substitution in the *ZRS* of *SHH*.^21^ Only two other patients in the cohort had triphalangeal thumb, and in neither case was the malformation bilateral; one patient had unilateral triphalangeal thumb associated with ipsilateral preaxial polydactyly and a mutation in *SALL1*,^22^ the other had triphalangeal thumb associated with tetralogy of Fallot, and no identified mutation. Thus, the presence of triphalangeal thumbs (especially if bilateral) is strongly suggestive of a mutation in the *ZRS* of *SHH*. Both patients with partial duplication of the ring finger had *HOXD13* mutations, but this criterion would miss three of the *HOXD13* mutations. Broadening the diagnostic criterion to syndactyly of the third webspace of the hand would yield two additional cases with *HOXD13* mutation, at the expense of including a further 18 subjects negative for *HOXD13* mutation.

### Estimation of the overall genetic contribution to CLM

In addition to the 23 probands in whom a genetic diagnosis was made, 39 other patients (25 with isolated postaxial polydactyly) had a family history of similar CLM, suggesting a contribution by either single gene mutations or polygenic variants. Two further sporadic patients had clinical diagnoses of Fanconi anaemia but did not have a specific mutation identified. Therefore, a minimum of 64/202 (32%) of patients with a CLM requiring reconstructive surgery have a genetic contribution to their malformation.

## Discussion

To our knowledge, this study is the first to screen systematically for mutations in an unselected cohort of individuals with CLMs requiring reconstructive surgery. We discovered mutations in *GLI3*, *HOXD13*, *SALL1*, *SALL4*, the *ZRS* of *SHH* and *TBX5* in 17 patients, karyotyping revealed a pathological rearrangement in a single patient, and the clinical genetics service independently obtained a cytogenetic or molecular genetic diagnosis in five patients, making a total of 23/202 (11%) patients in the cohort with a defined genetic diagnosis.

It may appear surprising that only 5/23 (22%) of these genetic diagnoses were achieved through routine clinical genetics services. This appears to reflect two factors. First, despite sometimes extensive family histories, most patients with genetic diagnoses (16/23) had not been referred by their medical carers for genetic counselling. It is possible that the relative lack of availability of genetic testing services for CLM, and a subjective lack of concern on the part of some parents about the nature and genetic implications of the CLM, may have contributed to this under-referral. Second, in the two additional cases previously referred to clinical geneticists, the (retrospectively correct) clinical diagnosis had not been confirmed molecularly. In one instance (OX2084, *TBX5* mutation) a tentative diagnosis of Holt–Oram syndrome (MIM 142900) had been made, but an electrocardiogram (ECG) and echocardiogram were normal, and genetic testing was not arranged. In another (OX3424, *ZRS* triplication), the correct clinical diagnosis of syndactyly type IV (Haas) (MIM 186200) was suggested, but the role of rearrangements of the *ZRS* of *SHH* in the aetiology of this disorder^25^ was not known at the time. In none of the probands with *GLI3* mutations had the diagnosis of Greig cephalopolysyndactyly (GCPS, MIM 175700) been suggested previously, even though this was clinically apparent retrospectively in four of the five subjects.

It is interesting to analyse the extent to which the universal screening approach that we adopted in this study fulfilled the three anticipated advantages that we identified in the introduction. Certainly we could estimate, in an unbiased fashion, the relative mutation frequencies of genes for which mutations have a well established role in CLM. Importantly, we did not identify any gene that is very commonly mutated in CLM; those genes most frequently mutated (*GLI3* and *HOXD13*, five cases each) demonstrated an overall prevalence of only 2.5–2.8%. Although the difference between identifying one and five cases in this series is not statistically significant, these data do support prioritisation of the introduction of testing for *GLI3*, *HOXD13* and the *ZRS* of *SHH* in clinical diagnostic services, on the basis that multiple mutations were identified: indeed, testing of the first two of these genes is now available through the Genetics Laboratories in Oxford (http://www.oxfordradcliffe.nhs.uk/forpatients/departments/labs/geneticslab/documents/diseaseservices.pdf (accessed 7 September 2009)). In many cases, careful evaluation of the phenotype would substantially enhance the specificity of genetic testing. For example, if the presence of bilateral preaxial polydactyly of the feet was made an essential criterion for testing *GLI3*, this would result in a sensitivity of 80% (four of five *GLI3* mutations identified) and a positive predictive value (PPV) of 100% (four of four individuals with specified phenotype have a *GLI3* mutation). A high sensitivity and PPV could also be achieved for the association of *ZRS* mutations and triphalangeal thumb (sensitivity 75%, PPV 60%). Mutations of *HOXD13* would be more difficult to pick out: although synpolydactyly 1 (SPD1; MIM 186000) is the classic phenotype associated with polyalanine tract expansion mutations (such as that identified in case OX2137), different molecular categories of *HOXD13* mutation present with variant phenotypes as illustrated by the other cases discovered in our series (table 2 and discussed below). Use of ring finger duplication or third web space syndactyly as the diagnostic criterion for *HOXD13* mutation in our series would have had good sensitivity (80%) but relatively poor PPV (18%).

A further benefit from our screening strategy is that we identified two new associations between CLMs and particular mutant alleles, both of which we have reported elsewhere: we found a novel *HOXD13* mutation encoding I314 L in two independent families segregating a specific disorder with features combining SPD1 and brachydactyly type E (MIM 113300),^18^ and a specific alteration of the *ZRS* of *SHH*, 295 T>C, as a common cause (three independent cases) of triphalangeal thumb, representing the mild end of a phenotypic spectrum including preaxial polydactyly type II (MIM 174500).^21^ In addition we found mutations or cytogenetic abnormalities in a further five cases that were not readily predictable from the phenotype (footnote † to table 2). Three of these (isolated postaxial polydactyly and *GLI3* mutation, unilateral preaxial polydactyly with triphalangeal thumb and *SALL1* mutation, limb reduction defect and t(2;18) chromosome translocation) have been reported in detail elsewhere.^22^ ^26^ The other two cases were preaxial polydactyly of the hands in a child with dup(6)(p22.3p23); and postaxial polydactyly of one foot with marked bilateral clinodactyly of the little fingers, associated with a heterozygous nonsense mutation (R319X) in *HOXD13* (supplemental fig S1D). Although we were not successful in identifying a novel role for any of the more speculative candidate human genes in isolated CLM, our DNA panel provides a resource for further genetic studies as new candidates are identified.

As part of this project we designed new MLPA kits for the identification of deletions in the *GLI3, HOXD13, ROR2, SALL1*, *SALL4* and *TBX5* genes. The diagnostic yield from MLPA analysis of the cohort was low, with only a single partial *GLI3* deletion being identified; however, the probe sets have subsequently been implemented in diagnostic laboratories, where additional deletions or duplications in *GLI3*, *HOXD13*, and *SALL1* have been identified in patients previously without a molecular diagnosis (M Oldridge, G Cross, personal communication, 2008).

Clearly the list of known CLM genes included in our screen was not exhaustive^27^; we focused on genes associated with variable CLM phenotypes in the better recognised, mostly dominantly inherited syndromes. Indeed some members of the cohort had clinical diagnoses (for example, Fanconi anaemia, ectrodactyly, and brachydactyly type C), that are associated with known genetic changes that we did not investigate, as this was outside the purpose of the study. These subjects were all included in the “genetic component” group in table 1. The minimum figure of 32% of patients from the cohort having a genetic aetiology for their malformation represents the first estimate of the genetic contribution to CLM. Previous epidemiological studies have focused on the incidence and type of malformation to aid in medical workforce planning,^1^ have focused on CLM diagnosed prenatally,^28^ or have looked for an association between environmental, maternal or teratogenic factors and specific types of CLM.^29^ Clinical features that predicted the discovery of a genetic cause for the CLM were the presence of a bilateral malformation, a positive family history of CLM, and an increasing number of limbs being affected. Furthermore, specific patterns of CLM predicted a genetic aetiology. Based on these data, we propose some simple guidelines (table 3) that should trigger the referral of patients by surgeons to clinical genetics services for diagnosis and investigation. All the suggested criteria at least double the likelihood of a specific molecular or cytogenetic abnormality being identified.

**Table 3:** Proposed criteria for referral of congenital limb malformation (CLM) to a clinical geneticist

	Number of cohort matching criterion	Cytogenetic or molecular diagnosis
**General criteria**		
1. Family history of CLM*	51 (25%)	12 (24%)
2. CLM associated with non-limb malformation	27 (13%)	10 (37%)
3. Severe CLM affecting more than one limb	47 (23%)	18 (38%)
**Specific criteria**		
4. Bilateral preaxial polydactyly of feet (*GLI3* mutation)	4 (2%)	4 (100%)
5. Ring finger duplication of hand (*HOXD13* mutation)	2 (1%)	2 (100%)
6. Bilateral triphalangeal thumb (*ZRS* of *SHH* mutation)	3 (1.5%)	3 (100%)

*Severe CLM excluded patients with post axial polydactyly, simple (non-bony) syndactyly, isolated clinodactyly, amniotic band sequence, and congenital trigger thumb.
